# Why do we need a new journal in autoimmunity?

**DOI:** 10.1186/1740-2557-1-1

**Published:** 2004-10-13

**Authors:** David D'Cruz, Vitaly Ablamunits

**Affiliations:** 1 The Rayne Institute, St Thomas' Hospital, London, UK; 2 St. Luke's-Roosevelt Hospital Center and Columbia University, New York, NY

## Abstract

A new online *Journal of Autoimmune Diseases *is created as an independent open access journal. In addition to the obvious advantages of the open access, the *Journal *will practice a double-blind reviewing of the manuscripts, which means that both the reviewers and the authors remain anonymous to each other. We believe that such a policy will reduce the influence of personal and other non-scientific factors on the reviewer's decision making.

## 

Autoimmune diseases are common and can affect virtually every organ in the body. They range from organ specific diseases such as thyroiditis or type 1 diabetes to life-threatening multi-system diseases such as systemic lupus erythematosus and the systemic vasculitides. Clinicians from every field of medicine may encounter these patients and both generalists and specialists need to keep up to date with clinical and experimental developments in autoimmunity. The advent of targeted therapies for example the anti-TNF-α agents for the treatment of rheumatoid arthritis exemplify the application of basic science research that can lead to effective therapies.

Inspired by the publisher, BioMed Central, a group of enthusiastic professionals gathered to launch the *Journal of Autoimmune Diseases*. There are several journals devoted to autoimmune diseases already, with at least four having the word "autoimmunity" in the title. In addition, a number of immunological journals and journals that specialise in particular diseases publish papers on autoimmunity. So is there a need for yet another journal?

The Editorial Board of *Journal of Autoimmune Diseases *certainly thinks so. This *Journal *is one of the new breed of online-only journals which are proving extremely successful. But it is not the magic word "internet' that makes the difference. Two distinct features provided by, BioMed Central and the Editorial Board offer special advantages to readers and authors.

## Open Access

The aim of publishing is to share information with the community. Authors are also keen to know that their work is being read. One of the syndromes of authorship has been the little card politely requesting a reprint. This card is sometimes forgotten or filed in the circular filing cabinet thus inducing considerable guilt both in the paper's author and the recipient. The era of reprint requests may be drawing to a close since most of the journals can now be found on-line. However, the access to a paper on-line often requires a subscription or a purchase of an individual article ("pay-per-view"). The high publication costs of printed journals deprive their publishers of the generosity of complete free access, but this tends to limit the dissemination of research. An alternative approach provided by BioMed Central is to make access totally free for everybody, as part of their Open Access policy [1]. It means that *Journal of Autoimmune Diseases *is universally and freely available online to everyone, its authors retain copyright, and it is archived in internationally recognised free repositories such as PubMed Central [2]; e-Depot [3]; Potsdam [4] and INIST [5].

*Journal of Autoimmune Diseases *enables scientists from countries and institutions with limited funds to read the same material as wealthier ones [6]. This has become possible because lower publication costs can be covered by the authors. The advantage for the authors is also obvious: access to their work is much more freely available throughout the world [7].

## Double-blinded peer review

The *Journal of Autoimmune Diseases *aims to provide a high standard of double-blinded peer review, in which the reviewer's name is not disclosed to the authors, and the authors remain anonymous to the reviewers. A few journals already use this system, so why do we believe that this will benefit the scientific community?

We have all come across situations where an honest, laborious piece of work was hard to publish. Some journals reject up to 95% of manuscripts [8], and this includes good papers. The problem here is that the decision made by a reviewer is not always based solely on the scientific merit of the paper. This may cause either of two antiscientific consequences, unfair rejection or unfair acceptance.

It is the nature of human beings that if a reviewer does not like you *personally*, a good paper is rejected or additional possibly unnecessary experiments are required which will take a year or two. Unfair rejection can occur due to a conflict of interest, be it personal or financial. For instance, an individual may be rejected on the basis of ethnicity [9], so may his paper be.

An unfair acceptance takes place when the reviewer is benevolent to a weak paper that has an outstanding name as the last author. Some authors may successfully exploit this weakness of a reviewer's human being by placing the famous name intentionally. This results in a ghost authorship of which the celebrity may be unaware! One of us remembers an anecdotal conversation he heard a few years ago in one of the universities:

Dorit (a secretary to the Professor): Robert? There is a new paper of yours I see on Medline. Do you wish me to update your publication list?

Professor: Who are the other authors?

(Dorit reads 20 last and first names)

Professor: Hm-m-m...I don't know any of these people. What is the title there?

(Dorit reads the title)

Professor: I don't remember even discussing anything like that or being consulted...

Dorit: Should I ignore it then?

Professor: Could you print it out for me? I will read it first. If the paper is good enough...Well, then I'll have nothing to do but to add it to my list of publications.

What are the remedies then? Two policies that could help to overcome these difficulties are completely open peer review and completely anonymous, or double-blind peer review. In open peer review [10], the reviewers' names are disclosed to the authors and vice-versa, ensuring accountability. Obviously, this might help against unfair rejections, but not against unfair acceptance. In addition, there is a danger that younger reviewers will be intimidated and the political power of the established will be increased [8]. We believe that a double-blinded review process will be much more effective in helping to avoid rejection of good papers and acceptance of unsound manuscripts for subjective, political or other non-scientific reasons.

### Journal of Autoimmune Diseases

We hope to attract clinical and basic science reports from leading and innovative authors. We call for established authors to publish with us in order to be easily accessible by all other scientists, to stop fighting with the windmills of the elite journals, to write detailed and clear papers in the unlimited space provided by internet. We would also strongly encourage more junior authors who are making their way in this field to consider publishing high quality science in the *Journal of Autoimmune Diseases *– we all need that important first paper to get going. The Editorial Board has been carefully selected from leading authorities in their own fields to help achieve our ambitious aim of developing a high class international journal that is accessible by all who have internet access and we look forward to receiving your submissions.

## References

[B1] BioMed Central Open Access Charter. http://www.biomedcentral.com/info/about/charter.

[B2] PubMed Central. http://www.pubmedcentral.org.

[B3] e-Depot. http://www.kb.nl.

[B4] Potsdam. http://www.uni-potsdam.de/over/homegd.htm.

[B5] INIST. http://www.inist.fr/index_en.php.

[B6] Tan-Torres Edejer T (2000). Disseminating health information in developing countries the role of the internet. BMJ.

[B7] Lawrence S (2001). Free online availability substantially increases a paper's impact. Nature.

[B8] Lawrence PA (2003). The politics of publication. Nature.

[B9] Watzman H (2003). Oxford professor accused of discrimination over e-mail. Nature.

[B10] Gura T (2002). Scientific publishing Peer review, unmasked. Nature.

